# Cadmium Levels in Urine and Mortality among U.S. Adults

**DOI:** 10.1289/ehp.11236

**Published:** 2008-09-03

**Authors:** Andy Menke, Paul Muntner, Ellen K. Silbergeld, Elizabeth A. Platz, Eliseo Guallar

**Affiliations:** 1 Department of Epidemiology, Johns Hopkins Bloomberg School of Public Health, Baltimore, Maryland, USA; 2 Department of Preventive Medicine, Mount Sinai School of Medicine, New York, New York, USA; 3 Department of Environmental Health Sciences, Johns Hopkins Bloomberg School of Public Health, Baltimore, Maryland, USA; 4 Department of Cardiovascular Epidemiology and Population Genetics, Centro Nacional de Investigaciones Cardiovasculares, Madrid, Spain

**Keywords:** cadmium exposure, cancer, cardiovascular disease, epidemiology, human, mortality, NHANES III

## Abstract

**Background:**

Cadmium exposure has been associated with increased all-cause, cancer, and cardiovascular disease mortality. However, studies investigating this association have included participants with considerably higher levels of cadmium than those found in the general population.

**Objective:**

We aimed to evaluate the association of creatinine-corrected urinary cadmium levels with all-cause and cause-specific mortality in the U.S. general population.

**Methods:**

We analyzed the relationship between cadmium measured in 13,958 adults who participated in the Third National Health and Nutrition Examination Survey in 1988–1994 and were followed through 31 December 2000, and all-cause, cancer, cardiovascular disease, and coronary heart disease mortality.

**Results:**

The geometric mean levels of urinary cadmium per gram of urinary creatinine in study participants were 0.28 and 0.40 μg/g for men and women, respectively (*p* < 0.001). After multivariable adjustment, including smoking, a major source of cadmium exposure in nonoccupationally exposed populations, the hazard ratios [95% confidence interval (CI)] for all-cause, cancer, cardiovascular disease, and coronary heart disease mortality associated with a 2-fold higher creatinine-corrected urinary cadmium were, respectively, 1.28 (95% CI, 1.15–1.43), 1.55 (95% CI, 1.21–1.98), 1.21 (95% CI, 1.07–1.36), and 1.36 (95% CI, 1.11–1.66) for men and 1.06 (95% CI, 0.96–1.16), 1.07 (95% CI, 0.85–1.35), 0.93 (95% CI, 0.84–1.04), and 0.82 (95% CI, 0.76–0.89) for women.

**Conclusions:**

Environmental cadmium exposure was associated with an increased risk of all-cause, cancer, and cardiovascular disease mortality among men, but not among women. Additional efforts are warranted to fully explain gender differences on the impact of environmental cadmium exposure.

Cadmium is a widespread toxic and carcinogenic metal (Agency for Toxic Substances and Disease Registry 1999; Verougstraete et al. 2003). In occupational studies, cadmium exposure has been associated with increased all-cause and cancer mortality, including lung, prostate, and renal cancer (Ades and Kazantzis 1988; Elinder et al. 1985; Pesch et al. 2000; Sorahan 1987). Population-based studies conducted in heavily polluted areas have linked cadmium to an increased risk of cancer, cardiovascular, and all-cause mortality (Arisawa et al. 2001; Nawrot et al. 2006; Nishijo et al. 1999, 2006). However, participants in these studies were exposed to relatively high levels of cadmium. The effects of cadmium exposure on mortality are not well characterized in general populations exposed to lower levels.

Environmental exposure to cadmium occurs primarily through smoking and the consumption of contaminated food and water (Satarug and Moore 2004; Verougstraete et al. 2003). Other sources include inhalation of contaminated air, particularly near specific point sources such as smelters and incinerators (Satarug et al. 2003). Cadmium accumulates in target tissues, especially in the renal cortex. The whole-body half-life for cadmium is estimated to be between 15 and 30 years (Bernard 2004; Satarug and Moore 2004).

The purpose of the present analysis was to evaluate the association of creatinine-corrected urinary cadmium with all-cause and cause-specific mortality in the general U.S. population using follow-up data from the Third National Health and Nutrition Examination Survey (NHANES III) Mortality Study. Urinary cadmium reflects cadmium concentrations in the kidneys, and it is considered the biomarker of choice to assess chronic exposure (Bernard 2004; Kjellstrom and Nordberg 1978).

## Materials and Methods

### Study population

NHANES III was a stratified, multistage probability survey designed to select a representative sample of the civilian noninstitutionalized U.S. population (National Center for Health Statistics 1994). Overall, 18,629 adults ≥ 20 years of age completed the NHANES III interview and examination between 1988 and 1994. We excluded 2,577 participants missing data for urinary cadmium, 105 participants missing data for urinary creatinine, 382 participants missing data for smoking history, 1,593 participants missing data for other covariates, and 14 who did not have follow-up information. After these exclusions, we included a total of 13,958 NHANES III participants in the present analyses. Participants we excluded due to missing data were older and more likely to be non-Hispanic black or Mexican American and to have a history of cardiovascular disease. However, those we excluded from the present study did not differ from those included in sex, smoking, or history of cancer. The protocol for NHANES III was approved by the National Center for Health Statistics of the Centers for Disease Control and Prevention Institutional Review Board. All participants gave written informed consent.

### Baseline data collection

NHANES III baseline data were collected during an in-home interview and a subsequent visit to a mobile examination center (National Center for Health Statistics 1994). During the in-home interview, demographic and health-related information was collected using a standardized questionnaire. We classified participants into one of three groups based on cigarette smoking: never smokers, former smokers, and current smokers. Smokers, both current and former, were also categorized into tertiles of pack-years smoked. Participants were considered to live in either a rural or an urban county based on U.S. Department of Agriculture rural–urban codes (National Center for Health Statistics 1994).

Height and weight were measured and body mass index (BMI) was calculated as weight in kilograms divided by height in meters squared. Blood pressure was measured three times during the in-home interview and three additional times during the participant’s visit to the mobile examination center. We averaged all blood pressure measurements for each participant and defined hypertension as systolic blood pressure ≥ 140 mmHg, diastolic blood pressure ≥ 90 mmHg, and/or current use of medication to lower blood pressure.

Serum C-reactive protein (CRP) was quantified using latex-enhanced nephelometry, a low-sensitivity assay. We classified participants as having CRP < 0.3 mg/dL (i.e., below the limit of detection), between 0.3 and 0.9 mg/dL, or ≥ 1.0 mg/dL. Plasma glucose was measured using an enzymatic reaction. We defined diabetes mellitus as fasting plasma glucose ≥ 126 mg/dL, nonfasting plasma glucose ≥ 200 mg/dL, and/or a self-reported history of diabetes with concurrent use of antidiabetes medication. Total serum cholesterol was measured enzymatically. Serum creatinine was measured by a kinetic rate Jaffe method. We calculated estimated glomerular filtration rate (eGFR) using the Modification of Diet and Renal Disease equation after aligning the serum creatinine concentrations with the assay employed in the development of the equation (Coresh et al. 2002; Levey et al. 1999). We classified individuals as having an eGFR of ≥ 90, 60–89, or < 60 mL/min/1.73 m^2^.

A detailed description of the measurement of urinary cadmium levels is available elsewhere (Gunter et al. 1996). In brief, all materials used for collecting and processing urinary cadmium specimens were screened for possible cadmium contamination. A spot urine specimen was collected and shipped at –20°C to the NHANES laboratory at the Centers for Environmental Health at the Centers for Disease Control and Prevention in Atlanta, Georgia (USA). Urinary cadmium was measured by graphite furnace atomic absorption with Zeeman background correction using the Centers for Disease Control and Prevention modification (Gunter et al. 1996) of the method of Pruszkowska et al. (1983). Specimens were analyzed in duplicate, and the average of the two measurements was reported. The detection limit was 0.03 μg/L.

The analytical laboratory followed extensive quality control procedures (Gunter et al. 1996). The National Institute of Standards and Technology standard reference material Toxic Elements in Freeze-Dried Urine (SRM 2670) was used for external calibration, with excellent agreement over the duration of the survey (Paschal et al. 2000). The interassay coefficients of variation ranged from 2.8% to 13.6%. Additionally, in an external quality assurance program from the Center of Toxicology of Quebec, laboratory measures were within 10% of reference means for urinary cadmium (*r*^2^ = 0.97) (Paschal et al. 2000).

Because NHANES III collected only spot urine samples, we performed all analyses using creatinine-corrected urinary cadmium values (urinary cadmium divided by urinary creatinine concentrations, expressed as micrograms per gram) to account for between-participant differences in urine dilution. Urinary creatinine was measured using the Jaffe method with a Beckman ASTRA automated analyzer (Paschal et al. 2000).

### Mortality follow-up

Adult NHANES III participants were followed for mortality through 31 December 2000. Matching was used to link NHANES III participants with the National Death Index to ascertain vital status and cause of death (National Center for Health Statistics 2006). Matching was based on 12 identifiers for each participant (e.g., Social Security number, sex, date of birth). A total of 1,690 eligible participants was identified as deceased over follow-up. Identical matching methodology applied to the NHANES I Epidemiological Follow-up Study (National Center for Health Statistics 2006) for validation purposes found that 96.1% of deceased participants and 99.4% of living participants were correctly classified.

We calculated follow-up for each study participant as the time between their NHANES III examination and the date of death, the date they reached 90 years of age, or 31 December 2000, whichever occurred first. We censored follow-up at 90 years of age because mortality was very high after this age and few participants contributed person-time experience in this age category. The mean follow-up time among participants who were alive at the end of follow-up and among participants who died was 9.0 years and 5.1 years, respectively.

In the NHANES III Mortality Study, cause of death was determined using the underlying cause listed on death certificates. The *International Classification of Diseases, 9th Revision* [ICD-9; World Health Organization (WHO) 1975], was used for deaths occurring between 1988 and 1998 and ICD-10 (WHO 1993) for deaths during 1999 and 2000. Cause-specific mortality was ascertained for cancer (ICD-9 codes 140–208, ICD-10 codes C00–C97), cardiovascular disease (ICD-9 codes 390–434 and 436–459, ICD-10 codes I00–I99), and coronary heart disease (ICD-9 codes 410–414 and 429.2, ICD-10 codes I20–I25).

### Statistical methods

Creatinine-corrected urinary cadmium levels were substantially different for men and women; moreover, we observed significant differences by sex in the association of cadmium with mortality. Therefore, we conducted all analyses separately for men and women. We categorized creatinine-corrected urinary cadmium levels in tertiles according to sex-specific cut points. We calculated baseline levels of study covariates for each tertile of creatinine-corrected urinary cadmium after adjusting for age (restricted quadratic spline with knots at the 10th, 50th, and 90th percentile of the overall study sample) and race/ethnicity (non-Hispanic white, non-Hispanic black, Mexican American, other) using linear regression for continuous covariates and logistic regression for dichotomous covariates.

For risk analyses, we used Cox regression to estimate the hazard ratios and 95% confidence intervals (CIs) for mortality associated with each tertile of creatinine-corrected urinary cadmium compared with the lowest tertile. We computed tests for linear trend across tertiles of creatinine-corrected urinary cadmium by including the median of each tertile as a continuous variable in the Cox regression models. Further analyses included creatinine-corrected urinary cadmium as a continuous, log-transformed variable. For these analyses, we present the hazard ratios for mortality end points associated with a 2-fold increase in creatinine-corrected urinary cadmium, which corresponds approximately to the difference between the 75th and the 50th percentiles of the creatinine-corrected urinary cadmium distribution in both males and females. We further evaluated the creatinine-corrected urinary cadmium–mortality relationships using restricted quadratic splines with knots at 0.086 μg/g, 0.378 μg/g, and 1.23 μg/g (corresponding to the 10th, 50th, and 90th percentiles of the creatinine-corrected urinary cadmium distribution in the overall study sample).

Cox models included adjustment for age (restricted quadratic spline with knots at the 10th, 50th, and 90th percentile of the overall study sample), race/ethnicity (non-Hispanic white, non-Hispanic black, Mexican American, other), menopausal status for women, urban residence (urban, rural), cigarette smoking category (never, former, current), tertile of pack-years smoked (< 9.0, 9.0–28.2, ≥ 28.3), alcohol consumption (< 12, ≥ 12 drinks in the past year), high school education, physical activity (none, 1–2, ≥ 3 times a week), annual household income (< $20,000, ≥ $20,000), BMI (continuous), CRP (not detectable, 0.3–0.9, ≥ 1.0 mg/dL), total cholesterol (continuous), diabetes mellitus, systolic blood pressure (continuous), use of antihypertensive medication, and category of eGFR (< 60, 60–89, ≥ 90 mL/min/1.73 m^2^). We tested for statistical interaction by including the product of creatinine-corrected urinary cadmium (log-transformed continuous) and each covariate in the Cox regression models. We conducted five separate sensitivity analyses *a*) excluding participants with a history of cardiovascular disease and/or cancer at baseline, *b*) adjusting for cotinine instead of pack-years smoked, *c*) adjusting for poverty index ratio as an alternative proxy for socioeconomic status (*n* = 12,930 with available data for poverty income ratio), *d*) adjusting for urinary creatinine in the Cox regression model instead of dividing cadmium concentrations by creatinine, and *e*) excluding participants with urinary creatinine ≥ 300 mg/dL. Results were qualitatively unchanged for each of these analyses (data not shown).

We analyzed data using SUDAAN (version 9.0; Research Triangle Institute, Research Triangle Park, NC, USA) to account for the complex NHANES sampling design, including unequal probabilities of selection, oversampling, and nonresponse.

## Results

The geometric mean creatinine-corrected urinary cadmium level was 0.28 μg/g in men and 0.40 μg/g in women (*p* < 0.001). Creatinine-corrected urinary cadmium levels were associated with age and with cigarette smoking in both men and women (Table 1). Higher creatinine-corrected urinary cadmium levels were also associated with a lower BMI, less than a high school education, no regular physical activity, household income < $20,000, a higher prevalence of CRP ≥ 1.0 mg/dL, and higher blood lead concentrations among men and women.

### Cadmium and mortality among men

**Figure 1:**
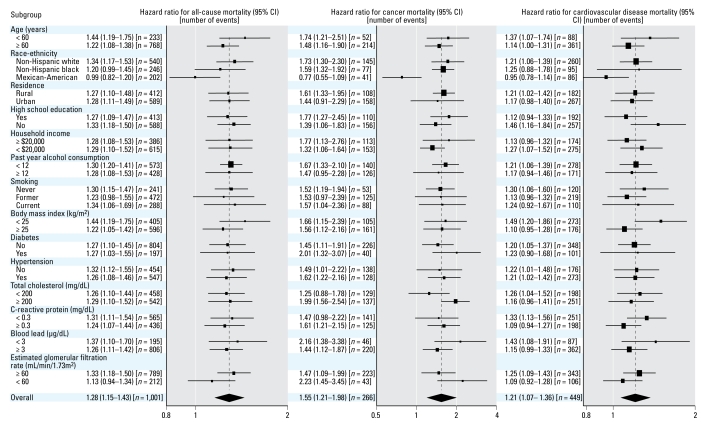
Multivariable-adjusted hazard ratio (95% CI) for all-cause, cancer, and cardiovascular disease mortality associated with a 2-fold increase in creatinine-corrected urinary cadmium overall, and by important subgroups among men. Adjustment included age (restricted quadratic spline with knots at the 10th, 50th, and 90th percentile of the overall study sample), race/ethnicity (non-Hispanic white, non-Hispanic black, Mexican American, other), urban residence (urban, rural), annual household income (< $20,000, ≥ $20,000), high school education, smoking category (never, former who quit ≥ 4 years ago, former who quit < 4 years ago, current), tertile of pack-years (< 9.0, 9.0–28.2, ≥ 28.2), physical activity (none, 1–2, ≥ 3 times a week), diabetes, BMI (continuous), alcohol consumption (< 12, ≥ 12 drinks in the past year), CRP (not detectable, 0.3–0.9, ≥ 1.0 mg/dL), total cholesterol (continuous), systolic blood pressure (continuous), blood pressure–lowering medication, blood lead (log-transformed), and eGFR (< 60, 60–89, ≥ 90 mL/min, 1.73 m^2^). A 2-fold increase in urinary cadmium corresponds approximately to the difference between the 75th and 50th percentiles of the cadmium distribution (0.61 μg/g and 0.32 μg/g, respectively). The sizes of the boxes are inversely related to the variance of the point estimate.

**Figure 2:**
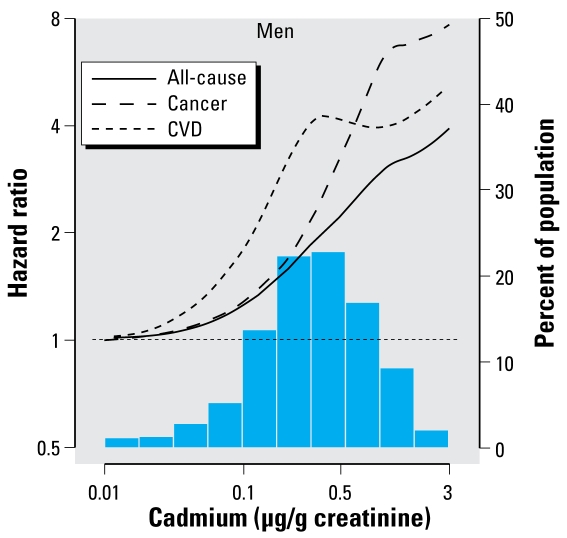
Multivariable-adjusted relative hazard of all-cause, cardiovascular disease, and cancer mortality associated with creatinine-corrected urinary cadmium using restricted quadratic splines among men (adjusted as listed for Figure 1).

The number of deaths due to all causes, cancer, cardiovascular disease, and coronary heart disease among men were 1,001, 266, 449, and 241, respectively. Among men, creatinine-corrected urinary cadmium levels were positively associated with increased mortality. The multivariable-adjusted hazard ratios for all-cause, cancer, cardiovascular, and coronary heart disease mortality for the highest compared with the lowest tertile were 1.68 (95% CI, 1.09–2.58), 4.29 (95% CI, 2.31–7.96), 1.33 (95% CI, 0.69–2.56), and 2.48 (95% CI, 0.85–7.27), respectively (Table 2). Because of few cancer deaths in the lowest tertile of creatinine-corrected urinary cadmium (*n* = 15), the hazard ratio estimates for cancer mortality comparing the upper tertiles to the lowest tertile may be unstable, although the two upper tertiles still showed a graded, positive association. The multivariable-adjusted hazard ratios for all-cause, cancer, and cardiovascular mortality associated with a 2-fold increase in creatinine-corrected urinary cadmium were 1.28 (95% CI, 1.15–1.43), 1.55 (95% CI, 1.21–1.98), and 1.21 (95% CI, 1.07–1.36), respectively (Figure 1). The analogous hazard ratio for coronary heart disease mortality was 1.36 (95% CI, 1.11–1.66). In spline regression models, the relative hazard for all-cause, cancer, and cardiovascular mortality in men increased throughout the range of creatinine-corrected urinary cadmium (Figure 2).

The association of creatinine-corrected urinary cadmium and mortality end points was consistent across selected subgroups of men except for Mexican Americans (*p*-value for interaction < 0.05 for all-cause, cancer, and cardiovascular mortality) (Figure 1).

### Cadmium and mortality among women

**Figure 3:**
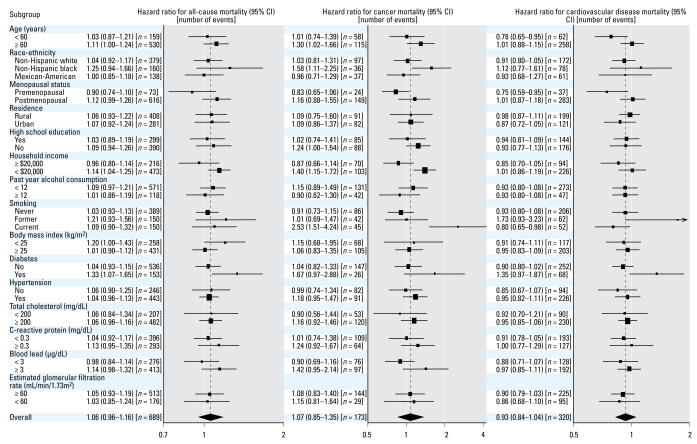
Multivariable-adjusted hazard ratio (95% CI) for all-cause, cancer, and cardiovascular disease mortality associated with a 2-fold increase in creatinine-corrected urinary cadmium overall, and by important subgroups among women. Adjustment included age (restricted quadratic spline with knots at the 10th, 50th, and 90th percentile of the overall study sample), race/ethnicity (non-Hispanic white, non-Hispanic black, Mexican American, other), postmenopausal status, urban residence (urban, rural), annual household income (< $20,000, ≥ $20,000), high school education, smoking category (never, former who quit ≥ 4 years ago, former who quit < 4 years ago, current), tertile of pack-years (< 9.0, 9.0–28.2, ≥ 28.2), physical activity (none, 1–2, ≥ 3 times a week), diabetes, BMI (continuous), alcohol consumption (< 12/≥ 12 drinks in the past year), CRP (not detectable, 0.3–0.9, ≥ 1.0 mg/dL), total cholesterol (continuous), systolic blood pressure (continuous), blood pressure lowering medication, blood lead (log-transformed), and eGFR (< 60, 60–89, ≥ 90 mL/min/1.73 m^2^). A 2-fold increase in urinary cadmium corresponds approximately to the difference between the 75th and 50th percentiles of the cadmium distribution (0.86 μg/g and 0.44 μg/g, respectively). The sizes of the boxes are inversely related to the variance of the point estimate.

**Figure 4:**
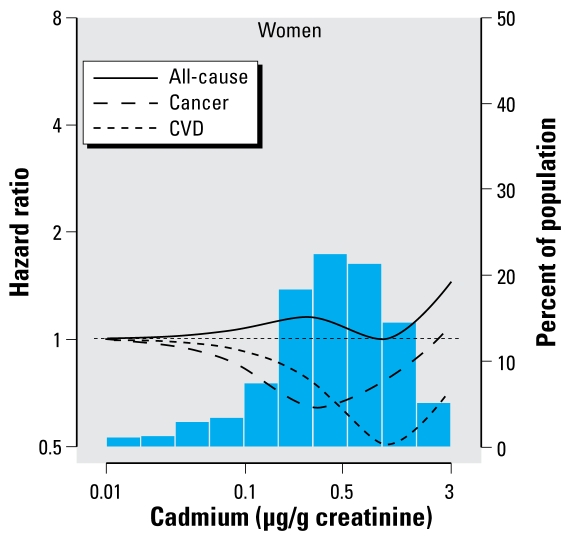
Multivariable-adjusted relative hazard of all-cause, cardiovascular disease, and cancer mortality associated with creatinine-corrected urinary cadmium using restricted quadratic splines among women (adjusted as listed for Figure 3).

The numbers of deaths due to all causes, cancer, cardiovascular disease, and coronary heart disease among women were 689, 173, 320, and 126, respectively. Among women, we found no association between creatinine-corrected urinary cadmium levels and mortality. The multivariable-adjusted hazard ratios for all-cause, cancer, cardiovascular, and coronary heart disease mortality for the highest compared with the lowest tertile were 1.14 (95% CI, 0.78–1.66), 1.11 (95% CI, 0.55–2.22), 0.82 (95% CI, 0.47–1.42), and 0.45 (95% CI, 0.24–0.83), respectively (Table 2). There are few cancer (*n* = 15) and coronary heart disease (*n* = 19) deaths in the lowest tertile of urinary creatinine-corrected urinary cadmium. Therefore, the hazard ratio estimates for cancer and coronary heart disease mortality may be unstable. The multivariable-adjusted hazard ratios for all-cause, cancer, and cardiovascular mortality associated with a 2-fold increase in creatinine-corrected urinary cadmium were 1.06 (95% CI, 0.96–1.16), 1.07 (95% CI, 0.85–1.35), and 0.93 (95% CI, 0.84–1.04), respectively (Figure 3). The analogous hazard ratio for coronary heart disease mortality was 0.82 (95% CI, 0.76–0.89). In spline regression models, we found no evidence of increased mortality with increasing creatinine-corrected urinary cadmium levels in women (Figure 4).

There were significant interactions of creatinine-corrected urinary cadmium and menopause status for all-cause and cancer mortality outcomes (*p*-values for interaction < 0.05), with higher hazard ratios among women who were postmenopausal than premenopausal (Figure 3). Additionally, creatinine-corrected urinary cadmium was positively associated with all-cause mortality among women with BMI < 25 kg/m^2^ (*p*-value interaction < 0.05) and with cancer mortality among non-Hispanic blacks, current smokers, and women with an annual household income < $20,000 (*p*-values for interaction < 0.05).

## Discussion

In this large, population-based prospective study, higher creatinine-corrected urinary cadmium levels were associated with increased all-cause, cancer, and cardiovascular disease mortality among men. This increased mortality was present among current, former, and never-smoking men, despite the differences in creatinine-corrected urinary cadmium levels among these three groups (geometric means of 0.42, 0.38, and 0.16 μg/g, respectively). Creatinine-corrected urinary cadmium was not associated with increased mortality among women overall, even though women had higher creatinine-corrected urinary cadmium levels than did men.

Our finding of higher creatinine-corrected urinary cadmium levels in women is consistent with previous studies showing a higher body burden of cadmium in women, regardless of the biomarker used (Nishijo et al. 2004). Furthermore, biopsy studies have demonstrated higher levels of kidney and liver cadmium in women compared with men (Benedetti et al. 1999; Satarug et al. 2002). This difference exists even though men are thought to have greater dietary intake of cadmium than did women (Choudhury et al. 2001). The reason for a higher body burden of cadmium in women seems to be related to their higher cadmium absorption in the digestive track. Cadmium is absorbed in the duodenum by the divalent metal transporter DMT1 (divalent metal transporter 1), the primary mechanism for uptake of nonheme iron that also has an affinity for cadmium (Nishijo et al. 2004). Iron deficiency up-regulates DMT1, increasing cadmium absorption. Therefore, women may absorb more cadmium through their digestive track than do men, on average, as a result of higher rates of iron deficiency (Vahter et al. 2007; Zacharski et al. 2000). Men and women may also differ in cadmium retention, regulated by metallothionein (Klaassen et al. 1999; Nordberg 2004). Metallothionein levels and expression of the metallothionein IIA gene (*MTIIA*) are higher in women than in men (Kwon et al. 2007), which could explain higher levels of cadmium in women as well as potentially greater protection against toxicity. Additionally, urinary cadmium concentrations seem to be genetically determined to a greater extent in women compared with men (Bjorkman et al. 2000).

Although higher cadmium levels in women point to sex differences in cadmium pharmacokinetics, it is unclear why cadmium was associated with mortality among men but not among all women in the present study. Few studies have presented mortality results for men and women separately. Among residents in a heavily polluted region of Japan (geometric mean creatinine-corrected urinary cadmium, 4.6 μg/g for men and 7.2 μg/g for women), there was a positive dose–response association between creatinine-corrected urinary cadmium and all-cause mortality over 15 years of follow-up among men and women (Nakagawa et al. 2006). However, in a separate contaminated region of Japan (median creatinine-corrected urinary cadmium, 7.0 μg/g for men and 12.1 μg/g for women), the hazard ratios for all-cause mortality, comparing residents with creatinine-corrected urinary cadmium ≥ 10 μg/g versus < 10 μg/g, were 1.83 (95% CI, 0.83–4.03) and 0.82 (95% CI, 0.43–1.59) for men and women, respectively (Arisawa et al. 2001). However, in this latter study, elevated urine β_2_-microglobulin, a biomarker of cadmium-induced renal tubular dysfunction, was associated with increased mortality among both men and women. Specifically, the hazard ratios for mortality comparing β_2_-microglobulin ≥ 1,000 μg/g of creatinine versus < 1,000 μg/g were 2.05 (95% CI, 0.94–4.47) and 2.05 (95% CI, 1.05–4.01) for men and women, respectively. Indeed, previous studies investigating the association of biomarkers of cadmium-induced renal tubular dysfunction with increased risk of mortality have consistently found associations among both men and women (Arisawa et al. 2001; Nishijo et al. 2006; Satarug et al. 2003).

Potential leads toward understanding the different association of cadmium and mortality in men and women may be derived from our observation of statistical inter action between cadmium and menopause status among women. This could result from a redistribution of cadmium from bone after menopause, which has been shown for lead (Nash et al. 2004). Cadmium is also stored in bone, although to a lesser extent than lead (Bocio et al. 2005). In addition, cadmium shows androgen- and estrogen-like activities *in vitro* and *in vivo*, with potential differential effects in premenopausal women, postmenopausal women, and men (Vahter et al. 2007). Further research is needed to confirm the heterogeneity of cadmium effects by sex and menopausal status at low levels of exposure and to identify the responsible mechanisms.

Cadmium and cadmium compounds are established carcinogens in humans (Agency for Toxic Substances and Disease Registry 1999; International Agency for Research on Cancer 1993). In the present study, higher creatinine-corrected urinary cadmium levels were associated with increased cancer mortality among men. This association persisted after adjusting for smoking status and pack-years of smoking. Lifetime cumulative smoking may not have been fully captured by our adjustment variables, and we cannot rule out residual confounding by smoking as an explanation of our findings. However, the association between creatinine-corrected urinary cadmium and mortality was remarkably similar when restricted to never-smoking men, making residual confounding by smoking less likely. It is important to note that the cadmium–cancer association may differ when investigating fatal cancer as opposed to all incident cancers. Our study included only cancer mortality, due largely to metastatic disease, a unique subset of all incident cancers.

Environmental exposure to cadmium has been found to be associated with cardiovascular disease mortality (Inskip et al. 1982; Nishijo et al. 2006). Conversely, higher levels of urinary and blood cadmium were not associated with prevalent cardiovascular disease in the CADMIBEL (Cadmium in Belgium) study (Staessen et al. 1991). One important difference between these studies is the distinction between cardiovascular disease mortality and all cardiovascular disease. Fatal cardiovascular disease is a unique subset of all incident cardiovascular disease. Therefore, the cadmium–cardiovascular disease association may differ when investigating all incident cardiovascular disease.

Cadmium may induce cardiovascular disease via renal damage (Bernard 2004; Satarug et al. 2006). Among 3,178 Japanese men and women, cadmium-induced renal tubular dysfunction was associated with a significantly increased risk of mortality due to heart failure, stroke, and kidney disease, with similar findings for men and women (Nishijo et al. 2006). The importance of cadmium-induced kidney damage in the U.S. general population, however, is uncertain, because signs of tubular damage are typically not detected until urinary cadmium concentrations are as high as 1.7 μg/g (Satarug et al. 2003). However, cadmium levels were associated with peripheral artery disease in the U.S. general population in NHANES 1999–2000 (Navas-Acien et al. 2004, 2005), suggesting that cadmium may play a role in the atherosclerotic process. Conversely, several studies conducted in Belgium investigating the association of urinary and blood cadmium with blood pressure found no indication that cadmium increases blood pressure among men or women (Staessen et al. 1984, 1988, 1991, 2000).

In addition to potential residual confounding by smoking, other limitations of our study include measurement error due to within-person variability in cadmium and creatinine excretion in single spot urine samples, and the limited study power to evaluate some causes of mortality. Also, in NHANES III, causes of death were assigned based on death certificates, which may contain inaccurate information and may not be generalizable to nonfatal events.

Despite these limitations, the present study has a number of strengths. NHANES III collected data on a variety of health exposures and outcomes using a rigorous protocol with extensive quality control procedures. NHANES III findings are generalizable to the U.S. noninstitutionalized civilian population (National Center for Health Statistics 1994). Finally, the large sample size allowed the investigation between creatinine-corrected urinary cadmium and mortality in key subgroups, after adjustment for important confounders, and after exclusion of participants with a history of cardiovascular disease or cancer at baseline.

In summary, increased creatinine-corrected urinary cadmium levels were associated with an increased risk of all-cause, cancer, and cardiovascular disease mortality among men in the U.S. general population. This association was present among most subgroups investigated, including non-Hispanic white and non-Hispanic black men, but not Mexican-American men. Furthermore, the association was present among men who reported never smoking, a group with relatively low levels of creatinine-corrected urinary cadmium. In contrast, higher levels of creatinine-corrected urinary cadmium were not associated with mortality among women. The potential public health implications of these findings are substantial, because exposure to low levels of cadmium is widespread. Future studies should be conducted to fully characterize the public health impact of environmental cadmium exposure and to explain the differences in cadmium-related mortality between men and women.

## Figures and Tables

**Table 1:** Baseline characteristics^a^ of study participants by sex and tertile of creatinine-corrected urinary cadmium.

	Men				Women			
	< 0.21 μg/g	0.21–0.47 μg/g	≥ 0.48 μg/g	*p*-Value	< 0.29 μg/g	0.29–0.67 μg/g	≥ 0.68 μg/g	*p*-Value
Characteristic	(*n* = 1,867)	(*n* = 2,173)	(*n* = 2,518)	for trend	(*n* = 2,115)	(*n* = 2,697)	(*n* = 2,586)	for trend
Age (years)	33.8 (0.6)	43.7 (0.6)	53.4 (0.4)	< 0.001	34.6 (0.4)	45.5 (0.7)	55.1 (0.7)	< 0.001
Non-Hispanic white (%)	83.1 (1.3)	75.0 (2.2)	74.6 (2.0)	0.005	81.5 (1.8)	75.1 (1.4)	73.2 (1.8)	0.007
Non-Hispanic black (%)	7.2 (0.6)	10.4 (0.7)	11.0 (0.9)	0.001	8.6 (0.9)	13.6 (1.0)	10.8 (1.1)	0.39
Mexican-American (%)	5.2 (0.5)	5.6 (0.6)	5.2 (0.5)	0.90	3.9 (0.4)	5.4 (0.5)	4.9 (0.4)	0.13
Postmenopausal status (%)	—	—	—	—	37.8 (1.6)	39.4 (1.5)	43.1 (1.6)	< 0.001
Urban residence (%)	52.6 (5.1)	51.3 (5.2)	42.7 (5.0)	0.01	48.3 (5.2)	48.0 (4.9)	46.5 (4.9)	0.52
Systolic blood pressure (mmHg)	124.7 (0.5)	124.2 (0.6)	125.1 (0.5)	0.32	120.5 (0.6)	120.2 (0.7)	119.6 (0.7)	0.29
Diabetes (%)	7.0 (1.4)	5.2 (0.6)	5.5 (0.4)	0.49	4.9 (0.9)	5.4 (0.8)	5.0 (0.4)	0.73
BMI (kg/m^2^)	27.1 (0.2)	27.0 (0.2)	26.0 (0.2)	< 0.001	26.4 (0.2)	27.1 (0.2)	26.0 (0.2)	0.006
Current smoker (%)	11.9 (1.1)	28.5 (1.1)	65.2 (1.6)	< 0.001	11.2 (0.9)	23.2 (1.3)	48.4 (2.0)	< 0.001
Pack-years ≥ 28 (%)^b^	3.6 (0.8)	13.3 (1.4)	43.6 (1.9)	< 0.001	1.7 (0.4)	5.4 (0.7)	24.2 (1.1)	< 0.001
eGFR < 60 mL/min/1.73 m^2^ (%)	4.5 (1.1)	3.7 (0.5)	3.5 (0.3)	0.25	4.6 (0.9)	6.2 (0.6)	4.6 (0.6)	0.16
High school education (%)	85.4 (1.5)	74.3 (1.6)	64.6 (1.9)	< 0.001	82.7 (1.4)	77.5 (1.4)	72.0 (1.5)	< 0.001
Consume alcohol (%)	67.9 (2.0)	65.9 (2.5)	67.9 (2.2)	0.74	43.5 (2.1)	45.0 (1.8)	42.5 (2.1)	0.44
No regular physical activity (%)	38.9 (1.6)	42.3 (2.1)	51.9 (1.5)	< 0.001	49.6 (2.8)	53.5 (1.3)	58.1 (2.1)	0.002
Household income < $20,000 (%)	21.6 (1.4)	26.8 (1.6)	39.6 (2.0)	< 0.001	29.7 (1.9)	34.9 (1.6)	41.2 (1.8)	< 0.001
CRP ≥ 1.0 mg/dL (%)	2.6 (0.6)	3.4 (0.5)	7.3 (1.0)	< 0.001	7.6 (0.7)	8.8 (1.0)	13.5 (1.1)	< 0.001
Total cholesterol (mg/dL)	199.9 (1.6)	201.9 (1.5)	204.3 (1.4)	0.05	208.9 (1.3)	205.0 (1.2)	204.1 (1.3)	0.01
Blood lead (μg/dL [geometric mean (95% CI)])	2.81 (2.61–3.04)	3.50 (3.28–3.74)	4.65 (4.38–4.94)	< 0.001	1.73 (1.63–1.84)	2.07 (1.97–2.17)	2.62 (2.45–2.81)	< 0.001

Values are means (SE) or percentages (SE) unless otherwise noted.

a All values except for age and race/ethnicity were standardized for age (restricted quadratic spline with knots at the 10th, 50th, and 90th percentile of the overall study sample) and race/ethnicity (non-Hispanic white, non-Hispanic black, Mexican American, other).

b This level represents the highest tertile of pack-years.

**Table 2:** Hazard ratio (95% CI) for all-cause, cancer, cardiovascular disease, and coronary heart disease mortality associated with sex and tertile of creatinine-corrected urinary cadmium.

	Men				Women			
Mortality	< 0.21 μg/g	0.21–0.47 μg/g	≥ 0.48 μg/g	*p*-Value for trend	< 0.29 μg/g	0.29–0.67 μg/g	≥ 0.68 μg/g	*p*-Value for trend
All-cause	*n* = 94	*n* = 250	*n* = 657		*n* = 71	*n* = 205	*n* = 413	
Age and race/ethnicity adjusted	1.00	1.54 (1.05–2.26)	2.48 (1.75–3.50)	< 0.001	1.00	1.32 (0.89–1.96)	1.66 (1.18–2.34)	0.005
Multivariable adjusted^a^	1.00	1.43 (0.95–2.16)	1.68 (1.09–2.58)	0.02	1.00	1.18 (0.78–1.78)	1.14 (0.78–1.66)	0.87
Cancer^b^	*n* = 15	*n* = 47	*n* = 204		*n* = 15	*n* = 46	*n* = 112	
Age and race/ethnicity adjusted	1.00	2.54 (1.35–4.80)	8.03 (4.58–14.1)	< 0.001	1.00	1.03 (0.51–2.08)	1.76 (0.88–3.50)	0.02
Multivariable adjusted^a^	1.00	2.16 (1.11–4.21)	4.29 (2.31–7.96)	0.001	1.00	0.93 (0.47–1.86)	1.11 (0.55–2.22)	0.52
Cardiovascular disease	*n* = 44	*n* = 129	*n* = 276		*n* = 41	*n* = 103	*n* = 176	
Age and race/ethnicity adjusted	1.00	1.75 (0.94–3.25)	1.89 (1.07–3.31)	0.03	1.00	1.06 (0.52–2.14)	1.05 (0.61–1.80)	0.92
Multivariable adjusted^a^	1.00	1.66 (0.89–3.11)	1.33 (0.69–2.56)	0.68	1.00	0.96 (0.47–1.97)	0.82 (0.47–1.42)	0.19
Coronary heart disease	*n* = 21	*n* = 65	*n* = 155		*n* = 19	*n* = 36	*n* = 71	
Age and race/ethnicity adjusted	1.00	2.58 (0.96–6.93)	2.58 (1.02–6.49)	0.15	1.00	0.56 (0.28–1.12)	0.69 (0.42–1.15)	0.81
Multivariable adjusted^a^	1.00	2.69 (0.94–7.71)	2.48 (0.85–7.27)	0.42	1.00	0.49 (0.26–0.90)	0.45 (0.24–0.83)	0.08

*n*, number of events.

a Adjustment included age (restricted quadratic spline with knots at the 10th, 50th, and 90th percentile of the overall study sample), race/ethnicity (non-Hispanic white, non-Hispanic black, Mexican American, other), postmenopausal status (among women), urban residence (urban, rural), annual household income (< $20,000, ≥ $20,000), high school education, smoking category (never, former who quit ≥ 4 years ago, former who quit < 4 years ago, current), tertile of pack-years (< 9.0, 9.0–28.2, ≥ 28.2), physical activity (none, 1–2, ≥ 3 times a week), diabetes, BMI (continuous), alcohol consumption (< 12, ≥ 12 drinks in the past year), CRP (not detectable, 0.3–0.9, ≥ 1.0 mg/dL), total cholesterol (continuous), systolic blood pressure (continuous), blood pressure–lowering medication, blood lead (log-transformed), and eGFR (< 60, 60–89, ≥ 90 mL/min, 1.73 m^2^).

b Because of few cancer (*n* = 15 for men and women) and coronary heart disease (*n* = 21 for men and *n* = 19 for women) deaths in the lowest tertile, the hazard ratio estimates for cancer and coronary heart disease mortality may be unstable.
